# Bioarchaeology and evidence of violence from a precolonial later stone age communal burial in South Africa

**DOI:** 10.1371/journal.pone.0310421

**Published:** 2024-09-17

**Authors:** Calvin G. Mole, Judith Sealy, Deano D. Stynder, Petrus J. Le Roux, Victoria E. Gibbon

**Affiliations:** 1 Department of Human Biology, Division of Clinical Anatomy and Biological Anthropology, University of Cape Town, Observatory, South Africa; 2 Department of Pathology, Division of Forensic Medicine and Toxicology, University of Cape Town, Observatory, South Africa; 3 Department of Archaeology, University of Cape Town, Rondebosch, South Africa; 4 Department of Geological Sciences, University of Cape Town, Rondebosch, South Africa; University of Padova: Universita degli Studi di Padova, ITALY

## Abstract

This study reports on the bioarchaeology and evidence of interpersonal violence in a group of archaeological skeletons found near Ladismith, Western Cape, South Africa. The co-mingled skeletal remains derive from at least ten individuals of varying ages and both sexes. Overlapping radiocarbon dates on three individuals place them in the first half of the 15^th^ century CE, pre-dating first European contact at the end of that century. Three juvenile crania have perimortem perforations, the locations of which indicate violent deaths. The sizes and shapes of the lesions suggest impact by a blade at least 110mm long and 50mm wide but with edges only 2mm thick. Based on these dimensions, we hypothesise that this was a metal-tipped spear. The nearest metal-working communities at this time lived approximately 500 km away, implying long-distance trade or exchange. δ^13^C, δ^15^N and ^87^Sr/^86^Sr values indicate that this was a heterogenous group of individuals who had spent their early lives in different locations and consumed varied diets, who had come together and were living in or travelling through the Ladismith area at the time of their deaths. This finding extends the timeframe and location for the practice of communal burial in the Holocene of southern Africa and provides additional support for the hypothesis that communal burials in this region tend to be associated with violence.

## Introduction

The exploration of conflict and violence in past and present societies is an important avenue of investigation [1]. Questions include the extent of inter-personal violence in the distant past, especially amongst hunter-gatherers compared with food producers, the scale (individual vs group coalition) and the likely causes of conflict, e.g. competition for resources, search for sociopolitical advantage [2–6].

Many small-scale societies have historically been viewed as inherently peaceful, however, there is growing literature suggesting significant levels of violence [2,3,7,8]. The approximately 20 000-year-old skeleton from the Egyptian site of Wadi Kubbaniya exhibits evidence of both healed and peri-mortem trauma [9,10], as does the larger assemblage of skeletal remains from Jebel Sahaba, in the Sudan, which are at least 13 400 years old [11]. It has been suggested that this stemmed from territorial disputes arising out of environmental pressures exacerbated by climatic instability [11]. Skeletal remains from Nataruk in the Turkana Basin, dated to around 10 000 years ago, have been interpreted as evidence of deliberate killing of a small group of foragers, a rare instance of inter-group (not just interpersonal) violence [4]. Stojanowski and colleagues [12] have, however, argued that some of the reported injuries were actually the result of taphonomic processes rather than blunt force trauma.

In southern Africa, the portrayal of recent hunter-gatherers as “harmless people” [7,13–15] is at odds with Lee’s [16] discovery of significant evidence of violence between 1920 and 1955 CE. Disruptions resulting from the activities of farmers and traders in the region may have played a role here [17]. The extensive skeletal record of Holocene Later Stone Age (LSA) people in southern Africa provides a perspective on prehistoric interpersonal violence in the region.

Until about 2000 years ago, southern Africa was occupied only by stone tool-using hunter-gatherers ancestral to the Kalahari San communities described by 20^th^ century anthropologists. Skeletal remains of these populations come mainly from the southern and western coasts of South Africa, and most date to the last 4500 years. Documented cases of interpersonal violence appear to cluster around 2500 to 2000 years ago [18–20] and may be linked to competition for food and other resources as human population numbers rose along the resource-rich coastline [18]. Recent re-assessment of radiocarbon dates [21] reveals greater contemporaneity than previously recognised with the appearance of domesticated animals shortly before 2000 BP [22]; this is unlikely to be mere coincidence. There are no wild sheep and cattle in southern Africa, so domesticated livestock came from regions to the north. There is a long-standing debate about whether these animals were brought by immigrants from outside the region, or whether populations already in the sub-continent acquired livestock from neighbouring communities to the north, without significant population movement [23–28].

Genetic sequencing of southern African populations descended from hunter/herders show a degree of admixture from a Eurasian-East African population [28–32], probably in the first millennium CE, although precise chronology has yet to be resolved. Some hunter-gatherer groups never took up herding and continued to hunt and gather for a living, while others may have acquired livestock but continued to hunt in times of need [24,33]. Herding would have placed pressure on hunter-gatherer groups in the region due to a decrease in game populations [34], and conflict may have arisen if livestock were hunted, as documented in early historic records [35,36].

**Fig 1:**
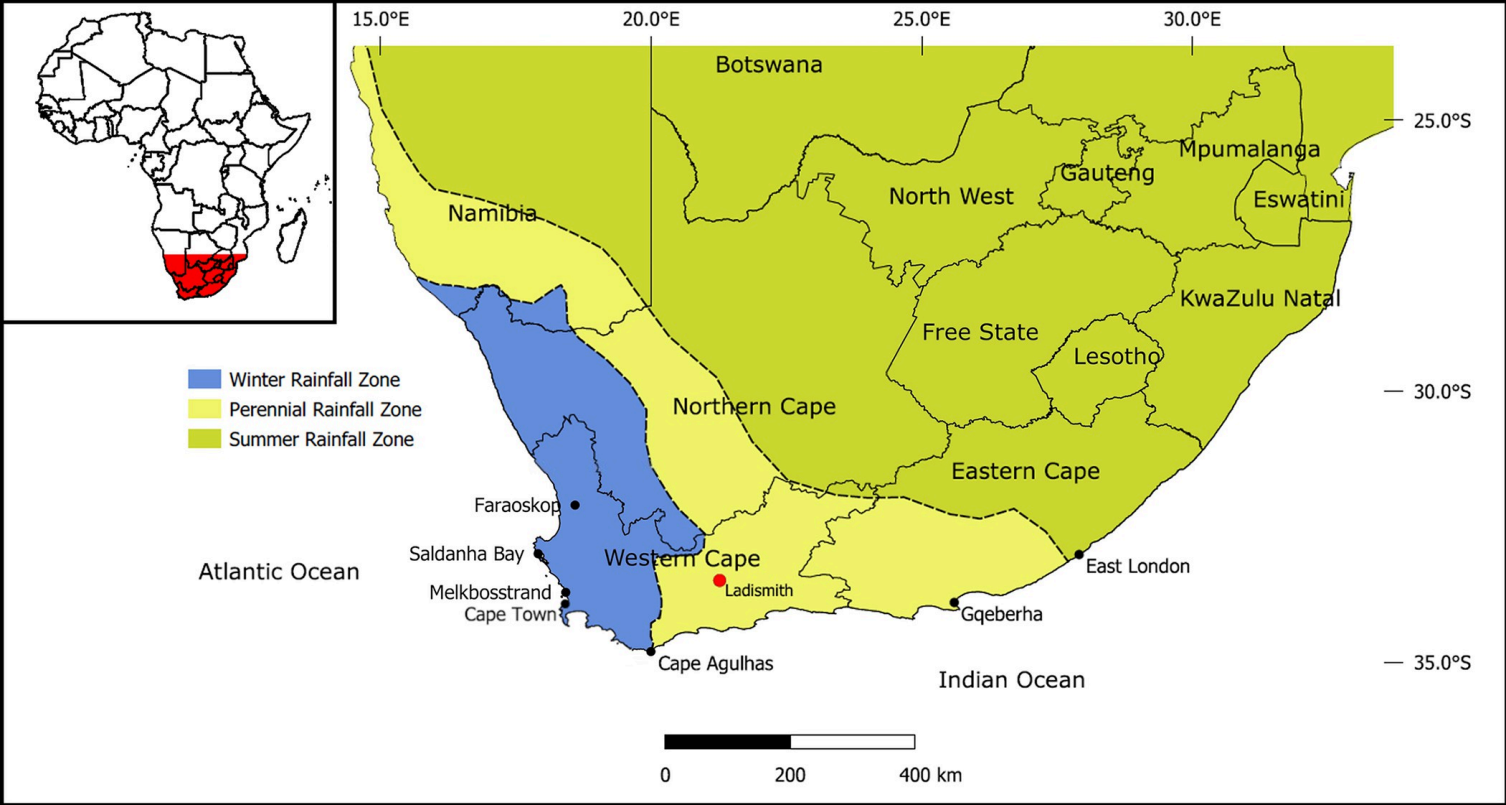
Map of southern Africa indicating location of Ladismith. Inset: Africa. Rainfall zones derived from Chase and Meadows [41]. Map produced in QGIS 3.16.16, geographical boundaries: Open Africa (https://africaopendata.org/).

From the end of the 15^th^ century CE, European ships visiting on the way to and from the East recorded aspects of the lifeways of indigenous populations at the Cape, and we have more detailed records after permanent Dutch settlement in the mid-17^th^ century CE [37,38]. At that time, herders with large numbers of cattle and sheep were the dominant social and economic groups in the Western Cape [39] (Fig 1). Hunter-gatherers, who occasionally provided services to herders as part of client-type relationships, formed an underclass that typically lived in marginal areas [40].

Herder societies had clearly defined rules of property inheritance, particularly for domestic stock [42, p.325-326]. People without stock were excluded from positions of power, and there were patron-client relationships in which “poorer” people acted as shepherds and suppliers of commodities to richer ones. Colonial period documents indicate that hunters often provided services to herders in exchange for food [40]. Theft of domestic stock frequently led to conflict between hunters and herders, sometimes escalating to warfare [17]. The patronage system contributed to the marginalisation of hunters by herders and was a major trigger of violence between the two groups [40].

While the skeletal record is the primary source of evidence for interpersonal violence in the period pre-dating written records [1], not all injuries leave traces on bones. Even within contemporary fatal assault cases, only 68% of victims have skeletal injury [43]. In non-fatal cases this is far lower [44–46]. Visible injuries on the skeleton cannot always be definitively associated with cause of death. Skeletal evidence for interpersonal violence amongst hunters and herders in this region has been summarised by Morris [19,20] and Pfeiffer [18]. Most individuals showing evidence of violence date to the period around or shortly before 2000 years ago (2300 BP– 1150 BP: calibrated ages) [21] and may be linked to the first appearance of herding in the region. The transition from hunting and gathering to herding seems to have been more disruptive than previously realised. A number of these burials indicate fatal violence against women and children, usually with penetrating and/or blunt force injuries to the cranium [47–49].

Communal graves containing three or more individuals are rare in the LSA of southern Africa [50], and generally appear to be associated with instances of violence [21,48]. At Faraoskop Rock Shelter, near Graafwater, 12 individuals dating to *c*. 2000 BP were buried with at least three displaying extensive cranial blunt force injury [20,51]. At Diaz Street, Saldanha Bay, six individuals also dating to *c*. 2000 BP were found buried in close proximity to each other. One female was found with a bone point, similar to those used as arrow tips, near her cervical vertebrae [52]. Further south, at Lagoon Beach, Milnerton, a grave containing four bodies included one adult female with perimortem cranial blunt injury [48]. Along the Modder River, Darling, three children dating from 2430–1940 cal BP [21] and exhibiting extensive perimortem cranial injuries were found buried together [49]. So far, there have been no reports of any graves containing three or more individuals without evidence of skeletal trauma.

Here we report on human skeletal remains discovered near the town of Ladismith, Western Cape Province (Fig 1). These include three crania that exhibit evidence of perimortem trauma. Our study entails an in-depth assessment of trauma, demography and diet to enhance the understanding of the history of these people. Detailed analyses of the three crania with perimortem damage offer insights into the possible circumstances surrounding their deaths. Two new radiocarbon dates were obtained, and δ^13^C, δ^15^N and ^87^Sr/^86^Sr values elucidate diet, patterns of mobility and degree of intra-group variation.

## Materials and methods

### Skeletal assemblage and site background

The skeletal assemblage, which consists of 108 individual skeletal elements, was recovered outside the town of Ladismith, Western Cape in July 1946 CE. Ladismith is located approximately 320 km (~199 mi) north-east of Cape Town (Fig 1). The climate is warm and dry, and the rugged, mountainous terrain is sparsely covered by a mosaic of succulents, fynbos shrubs and seasonal grasses. The region was occupied by hunters and, in the last 2000 years, also by herders, as attested by the extensive archaeological deposits at the nearby site of Boomplaas Cave [53,54]. Notes made at the time that the Ladismith skeletons were donated to the University of Cape Town (UCT) (S1 Fig), state that they derived from an antbear (aardvark; *Orycteropus afer*) hole (1.4 m deep, 1 m long, and just over 0.6 m wide) in the veldt (uncultivated land or bush) near Ladismith. Following collection, the individuals were formally donated to UCT by the Magistrate and Police of Ladismith, where they were subsequently accessioned in the Human Skeletal Repository of the Department of Human Biology.

The skeletal remains of these individuals have not previously been described in detail, although the dimensions of three of the juvenile crania and one adult were included in broader studies [55,56]. All necessary permits were obtained for the current study, which also complied with relevant regulations. Ethical clearance for this research was obtained from the University of Cape Town Human Research Ethics Committee (Ref: HREC 397/2021). Approval for radiocarbon dating and isotopic analyses was obtained from Heritage Western Cape (Case#: 20020612SB0413E).

### Biological profile estimation

Standard non-destructive and non-invasive methods were used for estimation of sex, age, and stature. Visual analyses were conducted using a magnifying lamp (3 diopter with 1.75x magnification), and measurements were carried out using Vernier callipers and osteometric boards.

Sex estimates were based on assessment of adult pelvic bones and crania, where these were preserved [57,58]. As biological sex in juveniles cannot be accurately estimated using morphometric or morphological traits, this was not attempted. The ages of juvenile individuals were estimated by assessing epiphyseal fusion and dental eruption stages [57,59,60]. To estimate the age of adults, cranial suture closure, cranial transition analyses, as well as auricular and pubic symphyseal surfaces of the pelvis were assessed where these elements were available [61–64]. Age-at-death for populations of short stature and low body weight, such as mobile South African hunter-gatherers, is often underestimated [65,66] and should be considered when interpreting the results. Stature was estimated using femoral length [67]. Assignation of skeletal elements to individuals was checked through visual pair matching, taking into account similarities in taphonomic degradation and age-related features in elements [68].

### Trauma analyses

Each bone was macroscopically assessed for the presence of traumatic lesions, mechanism of injury (blunt, sharp, perforating) and timing of injury (antemortem, perimortem, postmortem). Blunt force injury is due to a low velocity impact over a relatively large area, producing linear or radiating fracture lines, comminuted fractures, depressed fractures, and/ or crushed margins [69,70]. Sharp force injury is a result of shearing or compressive forces over a small area by a pointed or bevelled edge typically resulting in linear lesions with well-defined smooth margins, hinge fractures, slot fractures and/or bone polish [71]. Perforating injuries may be caused by blunt, sharp or projectile objects and are typically due to high energy impacts, that result in penetration of the cortical bone surface. Damage diagnostic of the injury mechanism should be viewed as a continuum rather than a set of discrete criteria: sharp objects with enough mass or impact force may produce injury with overlapping blunt characteristics (referred to as blunt-sharp) [72].

The timing of injury was assessed on characteristics of fracture surface margins. The presence of any evidence of healing, including callus formation or bone remodelling is indicative of injuries that occurred while the person was living (antemortem) [73]. Perimortem injuries are defined as occurring around the time of death. Fracture characteristics depend on the bone’s elasticity, which is influenced by its remaining collagen and moisture content. Depending on circumstances, bone may retain collagen and moisture for extended periods of time-after-death [74]. Therefore, perimortem refers to a period where bone reacts to loads as if it were fresh. Characteristics of perimortem injury include: plastic deformation; consistent colouring of fracture margins; smooth or undulating fracture margins; the presence of bevelling; oblique angles between fractures; adherence of bone fragments; and the presence of hinge fractures, flake defects, bone scales (small adhering bone flakes) or crushed margins [74–78]. In contrast, when bone is dry it behaves as a brittle material, characterised by extensive fragmentation with inconsistent colouring of fracture margins; rough or splintered fracture margins; perpendicular fracture angles; and the absence of bevelling.

Crania were scanned using computed tomography (CT) to aid in analysis of cranial injuries, using a Philips Brilliance CT scanner (Philips Brilliance 64; V2 6.2.21004) at the University of Cape Town Private Academic Hospital. The scan parameters were 120kV, 300mA and a slice thickness of 2 mm. Post processing and 3D rendering of CT data were conducted using 3D Slicer [79].

### Radiocarbon and other isotopic analyses

Radiocarbon dates were measured in the Oxford Radiocarbon Accelerator Unit (ORAU), Oxford University. Stable carbon and nitrogen isotope analyses were conducted in the Stable Light Isotope Laboratory in the Department of Archaeology, University of Cape Town. Strontium isotope analyses were conducted at the multi-collector inductively coupled plasma mass spectrometer (MC-ICP-MS) facility in the Department of Geological Sciences, University of Cape Town.

Carbon and nitrogen isotope ratios of bone and dentine collagen provide information regarding dietary composition at the time of collagen formation [80]. Sampling for isotopic analysis was constrained by the fact that all individuals are incomplete. One individual (UCT 152d) is represented solely by a mandible, others by only a few skeletal elements (see S2 Fig). It was therefore not possible to sample the same skeletal elements for each individual. The sampling strategy aimed to minimise damage to the assemblage, so as far as possible, targeted already fragmentary skeletal elements. Teeth were analysed (for both light isotopes and ^87^Sr/^86^Sr) only if they could be removed from the surrounding alveolar bone without damage. Details of the skeletal elements sampled are provided in Table 2.

For measurements of δ^13^C and δ^15^N in collagen, small samples of bone were removed using a Dremel hand-held saw with emery cut-off wheel. Where possible, samples were taken from already damaged areas. Small samples of dentine were cut from the root tips of easily removed teeth. To isolate collagen, samples were lightly abraded to remove surface contamination, then treated with 0.2M HCl at room temperature to dissolve bone mineral [81]. The acid was replaced every two days until the samples were soft and, in the case of bone samples, translucent. Samples were then rinsed three times with distilled water before being treated overnight with 0.1M NaOH to remove possible humic contamination. They were then soaked in distilled water, changed daily until the pH remained neutral, then freeze-dried. Approximately 0.45 mg of collagen was weighed into a tin cup and folded tightly to exclude air, then loaded into an automated Flash 2000 elemental analyser set to 1020°C. The resultant CO_2_ and N_2_ gases were swept in a stream of helium carrier gas into a Delta V Plus isotope ratio mass spectrometer via a Conflo IV interface. Internal laboratory standards (new Merck gelatine, ANU sucrose, chocolate and valine) were analysed with each run. Isotope measurements are expressed in delta notation in parts per mille (‰) calculated as δR = (Rsample/Rstandard-1)*1000 where R = the ratio of heavy/light isotopes of the element R. Carbon isotope values are reported relative to Vienna PeeDee Belemnite, and nitrogen relative to atmospheric nitrogen. The standard deviation of repeated measurements of laboratory standards was <0.2‰ for both δ^13^C and δ^15^N.

^87^Sr/^86^Sr in consumer tissues tracks values in the food and drink. Southern African hunters and herders consumed local products, so their ^87^Sr/^86^Sr values derive from the underlying geology of their area of residence. Near the coast, marine aerosols add strontium from seawater [82]. Sr isotope values in archaeological remains are usually measured in tooth enamel, which is less subject to post-depositional mineral alteration than bone. ^87^Sr/^86^Sr of enamel reflects area of residence at the time of tooth formation [83]. For measurements of ^87^Sr/^86^Sr in tooth enamel, whole teeth were placed in an Australian Scientific Instruments RESOlution SE laser ablation system (193 nm) with S155 ablation chamber coupled to a Nu Instruments NuPlasma high-resolution multi-collector inductively coupled plasma mass spectrometer, procedures modified after Le Roux et al. [84]. Three analyses were carried out for each tooth crown, each along a line 400 μm long parallel to, and as close as possible to the occlusal surface, the middle of the crown, and the dentine-enamel junction. Analysis paths were carefully chosen to avoid strongly curved surfaces, and damaged or defective areas of enamel. The line to be analysed was cleaned before ablation by sweeping the laser rapidly along it using a 64 μm laser spot at 50 μm.sec^-1^, followed by analysis at 2.5 μm.sec^-1^ using a 50 μm spot. The laser operated at 30 Hz with an energy density of 4 J.cm^-2^. Ablation was done using a sweep gas mixture in the sample chamber of helium (400 ml.min^-1^) and nitrogen (0.9 ml.min^-1^), later (prior to injection into the plasma) mixed with argon (0.95 L.min^-1^). A modern shark tooth was analysed repeatedly as an in-house reference, and yielded mean ^87^Sr/^86^Sr of 0.70900 (n = 6) in agreement with the ^87^Sr/^86^Sr value for modern seawater of 0.70917 [85,86], considering the long-term, 2 SD external reproducibility of this system is ± 0.0003 [84].

## Results

Osteological analyses of the skeletal assemblage indicated a minimum of ten individuals (Table 1). This is one more than the initial assessment by Drennan, who in 1947 accessioned the skeletal remains into the UCT Human Skeletal Repository, identifying a minimum of nine individuals under the designations UCT 148–150, UCT 151a-b, UCT 152a-c, and UCT 157. Reassessment of the individuals confirmed this assignment; however, an isolated mandible from an older individual, which could not be matched to any adult postcrania, was reclassified from UCT 152b to UCT 152d, increasing the total count to ten. Taphonomic assessment showed the skeletal elements are generally well preserved, with some root etching and sun bleaching. A complete skeletal inventory and images of the crania are provided in S2 and S3 Figs. Dental inventories are provided in S1 File. The adult cranium (UCT 157) and juvenile crania display characteristic San and/or Khoe morphology [87,88] (S3 Fig). In addition, the marked gracility of the bones and the short stature of UCT 152a, 152b, and 152c are characteristic of San and/or Khoe morphology [89].

**Table 1:** Summary of osteobiographical results for the Ladismith group.

Accession Number	Estimated sex	Estimated age (yrs)	Femur length (cm)	Estimated stature (cm)^a^	Trauma	Radiocarbon date
UCT 148	Indeterminate	14–16		-	-	
UCT 149	Indeterminate	7 ± 2		-	Perimortem, cranium	555 ± 18 BP (OxA-41315)
UCT 150	Indeterminate	6 ± 2		-	Perimortem, cranium	
UCT 151a	Indeterminate	5 ± 1.3		-	Perimortem, cranium and ulna	562 ±18 BP (OxA-41316)
UCT 151b	Indeterminate	12 ± 3		-	-	
UCT 152a	Male	40–44	L: 40.7 R: 40.05	154	Antemortem, ulna	
UCT 152b	Female	25–34	L: 39.1 R: 38.8	149	-	
UCT 152c	Male	35–39	L: 37.1 R: 36.95	143	-	
UCT 152d	Indeterminate	-		-	-	
UCT 157	Male	30–34		-	-	587 ± 28 BP (OxA-V-2055-45)

^a^ Estimated stature using Feldsman and Fountain [67] generic regression formula has a mean absolute deviation of 3.8 cm.

### Juvenile individuals

Five individuals are juveniles. One is an adolescent aged 14–16 years (UCT 148), three are children aged 5–7 years (UCT 149, 150, 151a), and an isolated mandible belongs to an individual aged approximately 12 years (UCT 151b). No unfused epiphyses, ribs, vertebrae or skeletal elements of the hands and feet are present for any of the children.

UCT 148 has a complete cranium and mandible, both scapulae, sacrum and most long bones. The right radius, right ulna and pelvic bones are missing. The proximal and distal humeral epiphyses are partially fused. Epiphyses of the leg bones are open. The following teeth are present: the left and right maxillary M1 and M2, right maxillary M3 crown, right mandibular M1, M2, M3 (crown) and left mandibular M2, M3 (crown). The fusion of epiphyses and pattern of dental eruption indicate this individual is between 14 and 16 years old.

UCT 149 has a complete cranium and mandible, right clavicle, both humeri, right ulna, and all long bones of the leg. All long bone epiphyses are open. Adult teeth present are unerupted except for the first molars; the right maxillary M1 and left mandibular M1 were lost post-mortem. Deciduous teeth present consist of left maxillary M1 and M2, left and right mandibular M1. The pattern of dental eruption and visible tooth crowns indicates this individual is 7 ± 2 years old.

UCT 150 has a complete cranium and mandible. The left radius and ulna, and both clavicles, humeri, femora and tibiae are present. All long bone epiphyses are open. All adult teeth present are unerupted. Deciduous teeth present consist of left and right maxillary M1 and M2, left maxillary cuspid, right mandibular M1, left and right mandibular M2. The pattern of dental eruption and visible tooth crowns indicates that this individual is 6 ± 2 years old.

UCT 151a has a complete cranium and mandible. Both humeri are present, but only the left radius and ulna, and left femur, tibia and fibula, all of which have open epiphyses. All visible adult teeth are unerupted. All deciduous maxillary teeth are present, except the right central incisor which was lost post-mortem. The left and right M1 and M2 are the only deciduous mandibular teeth present. The pattern of dental eruption and visible tooth crowns indicates that this individual is 5 ± 1.3 years old. UCT 151b consists of only an isolated mandible with left lateral incisor, canine, and P1; right P2; left and right M1 and M2 present. No deciduous teeth are present. The pattern of dental eruption indicates that this individual is 12 ± 3 years old.

### Adult individuals

There are five adults. UCT 152a, UCT 152b and UCT 152c are represented only by post-cranial elements. UCT 152a consists of a sternum, sacrum, both clavicles, scapulae and pelvic bones. All long bones are present except for the left ulna and right radius. UCT 152b has a sternal manubrium, right clavicle, right humerus, left radius, left ulna, left pelvic bone and all long bones of the legs. UCT 152c consists of a sternum, left scapula, right pelvic bone, and all long bones except for the left fibula. UCT 152d consists of an isolated mandible with only the left and right central incisors, left P1, M1, and M3 present. UCT 157 consists of a cranium and mandible with a sacrum and left pelvic bone; this is the only preserved adult cranium. The following teeth are present: left and right maxillary M1, M2 and M3, left maxillary canine, left and right mandibular M1, M2, M3 and P1. Ribs and skeletal elements of the hands and feet are absent in all adults.

### Presence of injury

With the exception of UCT 152a, who exhibits a well-healed callus on the distal portion of the right ulna, there is no evidence of prior injury or disease among any of the individuals. Perimortem perforating injuries to the neurocranium are observed in three children (UCT 149, UCT 150, UCT 151a). The penetrating lesions are detailed below. The morphology and locations of these injuries indicate that the children were impacted by a similar (or the same) weapon. The other two intact crania belong to an adolescent (UCT 148) and a young adult male (UCT 157), both of whom showed no evidence of perimortem injury.

**Fig 2:**
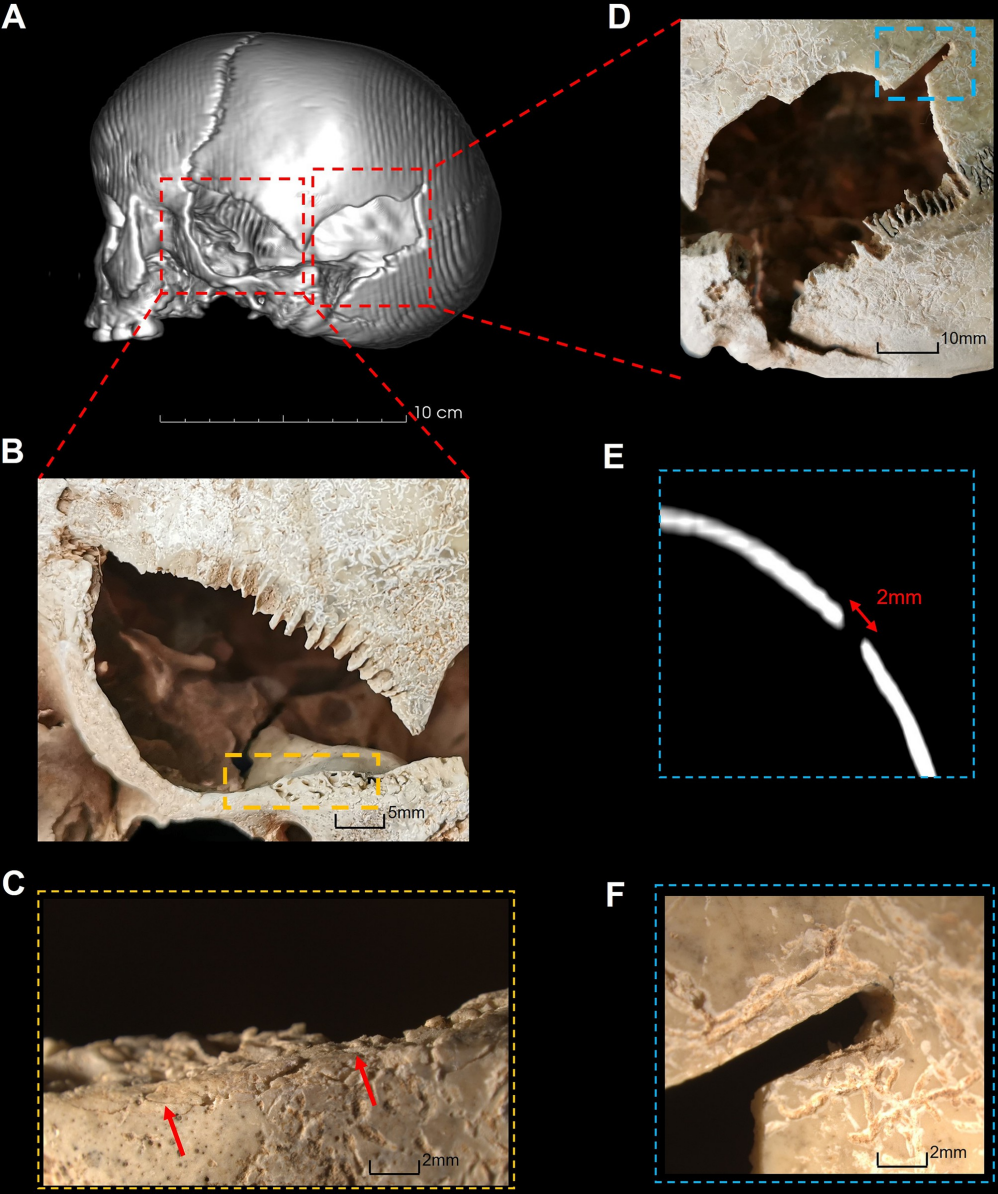
Cranial injuries on UCT 149. A: 3-Dimensional CT rendering of left-posterior cranium. B: Penetrating injury to left temporal region. C: Bone scales on inferior margin of B (red arrows). D: Penetrating injury to left parietal region. Blue box: Linear defect on superoposterior aspect of lesion. E: Measurement of linear defect on axial cross-section CT (2mm). F: Linear defect on superoposterior aspect of D.

**UCT 149.** UCT 149 displays evidence of perforations with two points of impact characteristic of sharp-blunt force injury and an associated blunt force injury on the opposite side of the cranium due to possible impact against a surface [20,90]. The fracture margins are uniform in colour and smooth with slight bevelling internally, indicating that the injury occurred perimortem. The two primary points of impact are located on the left lateral region of the neurocranium involving the squamosal portion of the temporal bone and the posterior aspect of the parietal respectively (Fig 2). The irregularly shaped defect to the squamosal region measures 36 mm x 52 mm (Fig 2B). The superior margin is defined by the parietal border of the squamosal suture, and the anterior margin of the defect involves the mid-portion of the greater wing of the sphenoid. The inferior margin is straight-edged, with bone scales (arrowed in Fig 2C) indicating that the impact was in an anterior-medial direction. This impact caused separation of the left and superior regions of the coronal suture and extended separation of the spheno-squamosal suture into the anterior base of the skull resulting in bone loss lateral to the left foramen ovale.

The defect on the left posterior parietal measures 45 mm x 30 mm (Fig 2D). The anterior margin runs superiorly, posterior to the left mastoid with the inferior margin running along the lambdoid suture. A radiating fracture extends 22 mm inferiorly into the occipital bone from the most inferior point of the defect. The superior margin displays straight-edged margins with a well-defined linear defect extending from the superoposterior aspect. This linear defect has a uniform width measuring 2 mm, extending 12 mm in a posteromedial direction (Fig 2E and 2F). The injury to the left parietal was caused by two impacts in an anteromedial direction.

The right zygomatic arch is fractured with associated separation of the right spheno-squamosal suture and partial separation of the right zygomatico-frontal suture. Separation of the spheno-squamosal suture extends into the cranial base resulting in loss of an 8 mm x 24 mm portion of bone, the medial aspect of which bisects the right foramen ovale. This is interpreted as injury resulting from the right anterior portion of the cranium being crushed against a hard surface at the time of the left-hand side impacts [20,90]. These injuries are consistent with the child lying on their right-hand side, perhaps on the ground, during impact.

### UCT 150

**Fig 3:**
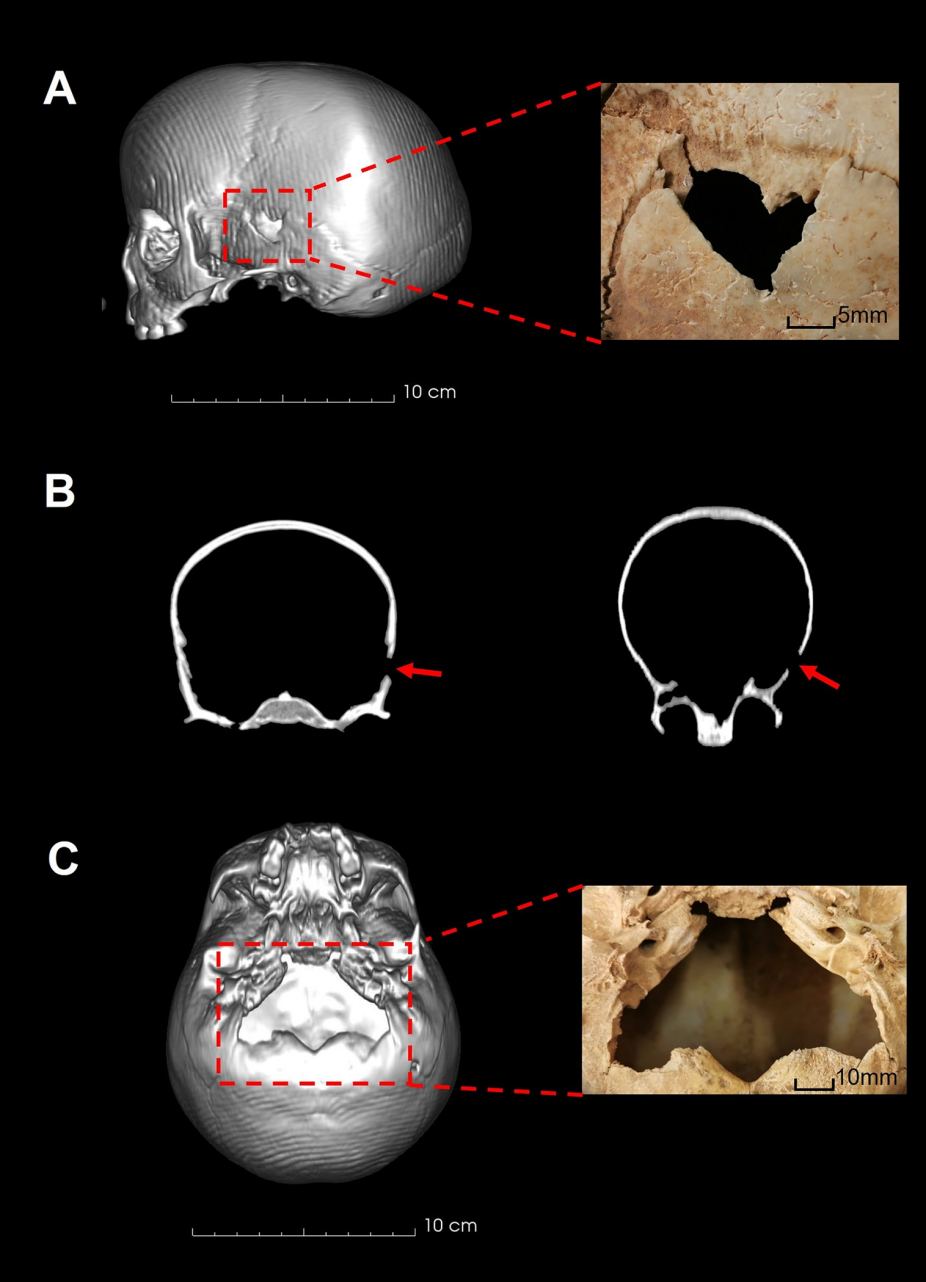
Cranial injuries on UCT 150. A: 3-Dimensional CT rendering of left cranium. Red box: Penetrating lesion to the left temporal region B: Coronal and axial cross-section CT demonstrating injury location and impact direction. C: 3-Dimensional CT rendering of inferior cranium. Red box: Penetrating lesion to the basal portion of the occiput.

UCT 150 has two perforating injuries to the neurocranium with characteristics of sharp-blunt force injury (Fig 3). A triangular-shaped defect is present on the left squamosal portion of the temporal bone situated on the anterior-superior border of the squamosal suture and spheno-squamosal suture (Fig 3A). The anterior aspect of the defect has a well-defined straight-edged margin measuring 15mm in length with slight bevelling present internally. The posterior margin of the defect has an undulating fracture margin of 11 mm in length. The anterior and posterior fracture margins intersect inferiorly with a notch defect measuring 2 mm wide with evidence of some bone flaking. The superior margin of the defect is formed by the inferior border of the parietal bone and measures 20 mm long. No associated radiating fractures are present. The uniform colour, smooth and undulating facture margins and presence of bone flaking indicate the injury occurred perimortem. A perimortem fracture to the left zygomatic arch and slight separation of the spheno-squamosal suture is associated with this impact, corresponding with a single impact directed medially.

In addition, a large irregularly shaped blunt defect (62 mm x 44 mm) is present on the cranial base involving the entirety of the basal portion of the occiput, anterior to the posterior margin of the occipital condyles (Fig 3C). The lateral margins of this defect run along the occipitomastoid sutures. The posterior fracture margin displays evidence of bone flaking on the external surface. The uniform colour, undulating facture margins and presence of bone flaking indicate that the injury occurred perimortem. This injury was likely caused by a single blow directed in a superior-anterior direction, applied to the posterior neck and head near the foramen magnum. This injury is consistent with a direct impact wherein the child was standing or kneeling, and the head tilted forward during impact [91,92].

### UCT 151a

**Fig 4:**
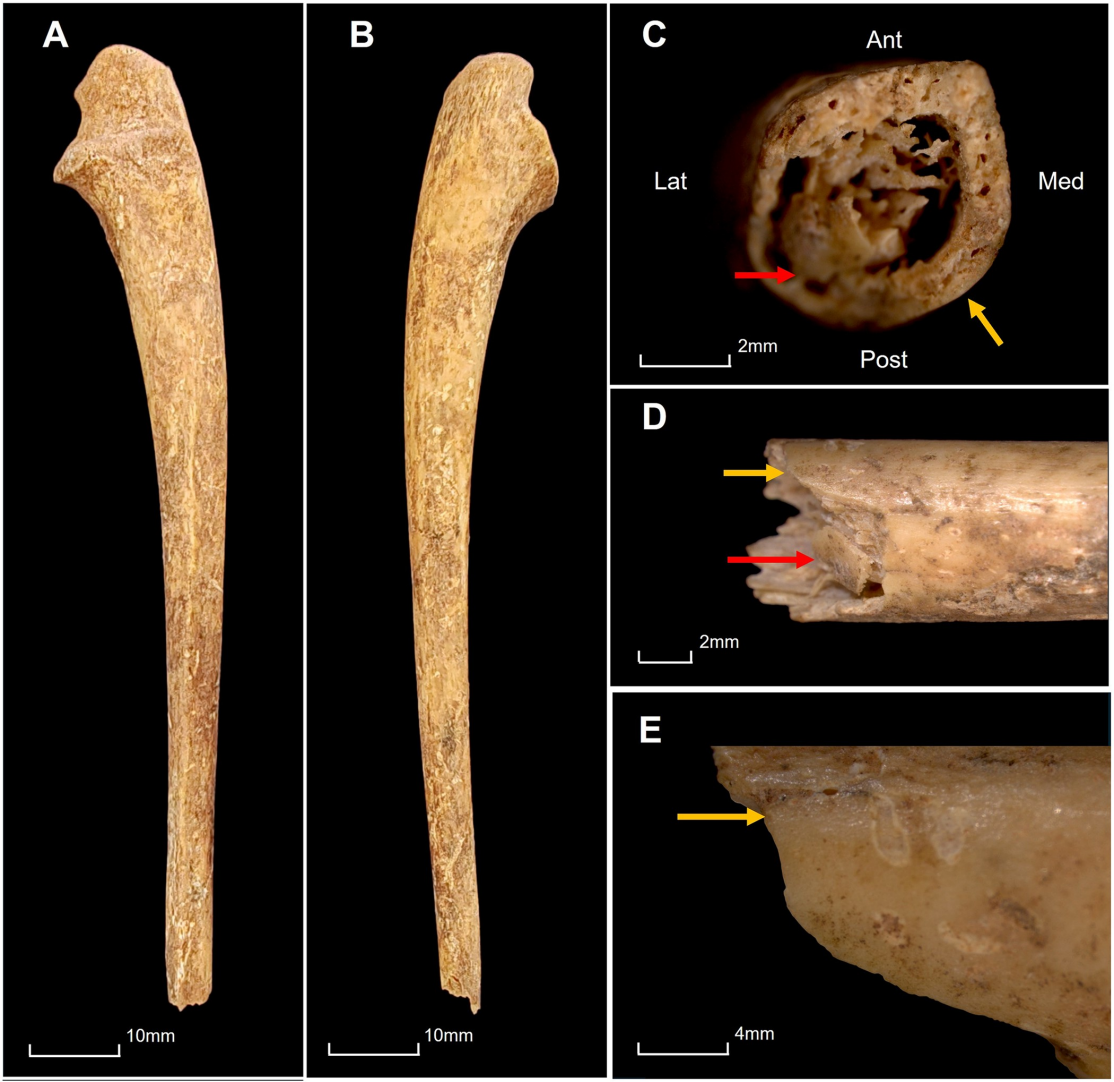
Left Ulna of UCT 151a. A: Medial view. B: Lateral view. C: Fracture surface demonstrating uniform colour. D: Posterior view of fracture margin. E: Posteromedial view of fracture margin. Yellow arrow: perimortem fracture margin. Red arrow: adhering bone fragment.

**Fig 5:**
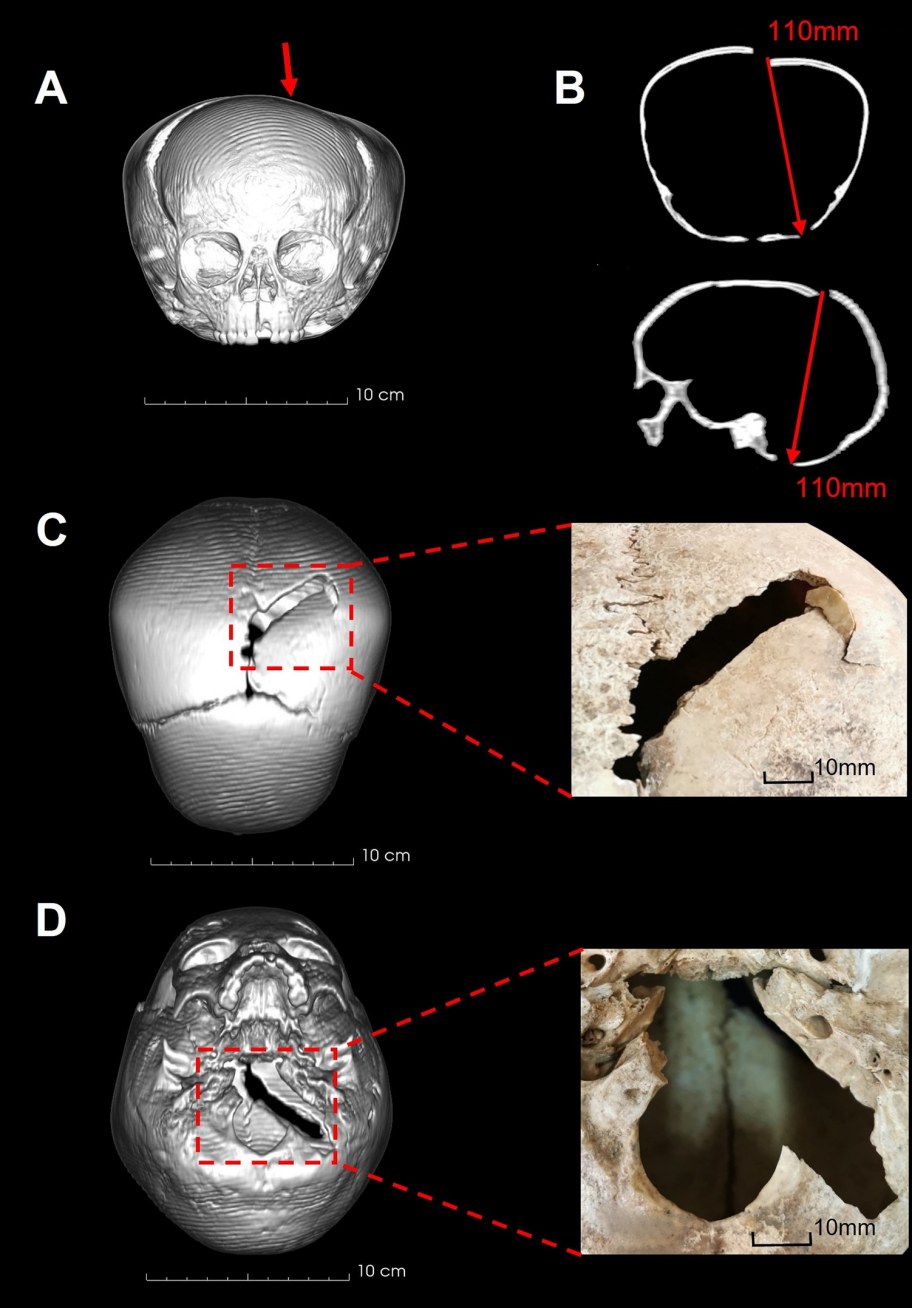
Cranial injuries on UCT 151a. A: 3-Dimensional CT rendering of anterior cranium demonstrating cranial deformation. Red arrow: Impact direction and location of superior penetrating lesion. B: Coronal and sagittal cross-section CT demonstrating direction of penetration and distance between superior and inferior lesions. C: 3-Dimensional CT rendering of superior cranium. Red box: Penetrating lesion to the left parietal bone. D: 3-Dimensional CT rendering of inferior cranium. Red box: Penetrating lesion to the middle and left posterior cranial base.

The third child, UCT 151a, has a transverse fracture of the left distal ulna shaft (Fig 4) and two perforating injuries to the superior neurocranium and cranial base respectively, with characteristics of sharp-blunt force injury (Fig 5). The fracture surfaces and surrounding bone are uniform in colour. The superior fracture retains a large, indented bone fragment on the posterolateral margin. Both characteristics indicate the injury occurred perimortem. The surface morphology of the ulna fracture indicates a region of tension on the posteromedial surface of the ulna (Fig 4E), consistent with this fracture being a defensive injury [93].

Both cranial lesions are rectangular in morphology. The superior lesion measures 5 mm x 50 mm and is situated on the superior aspect of the left parietal bone, at an angle of 50° to the sagittal plane (Fig 5C). The medial border of this lesion is 22 mm posterior to the bregma, along the sagittal suture. The anterolateral fracture margin has a well-defined straight edge with bone adherence on the lateral aspect of the lesion associated with a semi-lunar radiating fracture extending 16 mm anteriorly. The posteromedial fracture margin has an undulating surface. Associated with this injury is plastic deformation of the cranial vault with the left parietal bone deformed inwards toward the brain and lateral displacement of both the left and right parietal bones along the coronal suture. The uniform colour, smooth and undulating facture margins, and presence of plastic deformation indicate that the injury occurred perimortem.

The inferior lesion measures 12 mm x 55 mm and is situated diagonally across the middle and left posterior cranial base, at an angle of 50° to the sagittal plane (Fig 5D). The posteromedial border of the lesion has a well-defined straight edge that transects the foramen magnum at the posterior margin of the left occipital condyle and extends 28 mm in a posterolateral direction into the occipital bone. The anterolateral margin of the injury has an undulating surface and runs along the left occipitomastoid suture, extending across the left petrous bone.

The alignment and similarity of shape between the superior and inferior lesions indicate that both are due to a single impact directed from above, slightly to the left and anteriorly, which penetrated the entire cranium. As with UCT 150, these injuries are consistent with a violent impact with the perpetrator standing directly above the child and driving the implement downward through the cranium. The basicranial injury was not caused by a blow from below, which would require almost vertical penetration through the vertebrae and neck muscles.

### Isotopic analyses

**Fig 6:**
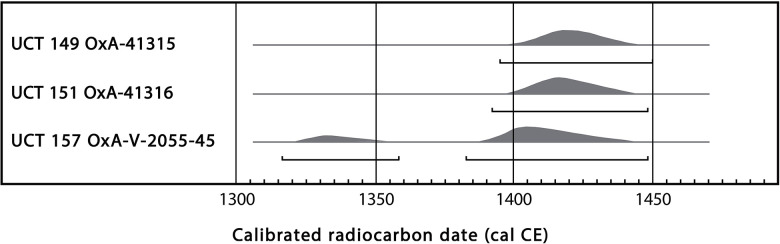
Calibrated age ranges for radiocarbon dates on UCT 149, 151a and 157, using OxCal version 4.4 [95] and calibration curve SHCal20, based on atmospheric data of Hogg et al.[96]. Brackets show 3 sigma age ranges, i.e. there is 99.7% likelihood of true ages falling within these ranges.

There are radiocarbon dates for three individuals. One date has been previously published: UCT 157 (587 ± 28 BP, OxA-V-2055-45) [94], and two are newly acquired: UCT 149 (555 ± 18 BP, OxA-41315), and UCT 151a (562 ± 18 BP, OxA-41316). As shown in Fig 6, the calibrated age ranges fall mostly in the first half of the 15^th^ century CE, pre-dating the arrival of the first European ships at the southern tip of Africa (in 1487–1488).

Bone and dentine collagen all yielded %C, %N and C:N values indicative of well-preserved collagen. They meet the collagen quality criteria proposed for archaeological samples [97], with C:N values also falling within the narrower range (3.0–3.3) proposed for contemporary samples [98]. The isotope ratios measured on this material are therefore likely to be reliable.

δ^13^C values range from -16.9 ‰ to -13.1 ‰ and δ^15^N from 10.3 ‰ to 13.4 ‰ (Table 2). These values are best interpreted in comparison with similar analyses of other archaeological skeletons from this region (see discussion; Fig 8). Individuals with more positive δ^13^C and high δ^15^N values consumed a greater proportion of marine foods, whereas those with low δ^13^C and δ^15^N consumed mainly terrestrial foods [99]. For one individual, UCT 148, collagen was analysed from both bone (blade of scapula) and dentine (*c*. 2 mm of root tip of left mandibular M2). The roots of mandibular second molars complete their growth at 14.5 to 17.5 years [100]. Since this individual died at the age of 14–16 years (Table 1), this tissue formed very shortly before death. δ^13^C values were -15.3 ‰ and -16.7 ‰ and δ^15^N 10.8 ‰ and 12.1 ‰ respectively. Values for dentine are likely to reflect diet closer to the time of death. The scapula, would have been growing rapidly in a 14–16 year old, and would also have included tissue formed over a period of at least several years [60]. Differences of 1.3 ‰ to 1.4 ‰ are substantially larger than analytical error, but have been reported for different individuals consuming the same diet [101,102].

**Table 2:** Isotope results and collagen quality indicators. Values for bone shown in ordinary type, dentine in italics and underlined. DEJ: Dentine-enamel junction.

		Bone / *dentine*						Enamel ^87^Sr/^86^Sr				
Accession number	Skeletal element sampled	UCT Lab #	δ^13^C (‰)	δ^15^N (‰)	Wt % C	Wt % N	C/N	Occlusal	Middle	DEJ	Range	Mean
												
UCT 148	Blade of left scapula		-15.3	10.8	42.7	15.6	3.2					
UCT 148	Left mandibular M2, roots complete	*24564*	*-16*.*7*	*12*.*1*	*42*.*6*	*15*.*5*	*3*.*2*	0.71962	0.71960	0.71995	0.00035	0.71972
UCT 149	Humerus shaft		-16.7	11.5	43.0	15.6	3.2					
UCT 149	Left maxillary M1							0.71483	0.71533	0.71496	0.00050	0.71504
UCT 150	Distal tibia (cortical + cancellous bone)		-13.1	11.1	42.6	15.4	3.2					
UCT 151a	Femoral shaft		-15.1	13.4	43.4	15.7	3.2					
UCT 151b	Left mandibular M2, dentine forming at time of death	*24565*	*-14*.*8*	*12*.*2*	*42*.*5*	*15*.*5*	*3*.*2*	0.71766	0.71725	0.71764	0.00041	0.71752
UCT 152a	Outer part of right femoral mid-shaft		-14.9	12.4	42.7	15.5	3.2					
UCT 152b	Radius shaft fragment		-13.5	10.6	43.4	15.6	3.3					
UCT 152c	Radius shaft fragment (larger radius than 152b)		-16.7	11.0	42.5	15.5	3.2					
UCT 152d	Left mandibular M3	*24566*	*-16*.*9*	*10*.*3*	*47*.*7*	*17*.*3*	*3*.*2*	0.71110	0.71175	0.71173	0.00065	0.71153
UCT 157	Left mandibular P1							0.71883	0.71820	0.71919	0.00099	0.71874
UCT 157	Left maxillary canine	*24567*	*-15*.*9*	*13*.*4*	*42*.*6*	*15*.*3*	*3*.*2*	0.71868	0.71888	0.71761	0.00127	0.71839

† Dentine samples all obtained from root tips.

Strontium isotope analysis (^87^Sr/^86^Sr) of tooth enamel was possible only for UCT 148, 149, 151b, 152d and 157, for whom permanent teeth were preserved. Each tooth was analysed three times: near the occlusal surface, in the middle of the crown and near the dentine-enamel junction. Results are reported in Table 2. Mean ^87^Sr/^86^Sr per tooth ranged from 0.71153 for UCT 152d to 0.71972 for UCT 148. Intra-tooth ranges for UCT 148, 149, 151b and 152d were 0.00035, 0.00050, 0.00041 and 0.00065 respectively. These ranges are relatively small, especially considering that analytical uncertainty on these measurements is 0.0003 (two sigma). Intra-tooth ranges for UCT 157 were larger: 0.00099 for the left mandibular first premolar, and 0.00127 for the left maxillary canine; across the two teeth the range extends from 0.71761 to 0.71919, a difference of 0.00158. The implications of this variation are discussed in the next section.

## Discussion

This study explores aspects of violence, diet and demography within a group of people who died in the region of the present-day South African town of Ladismith. These people are of interest because it is very unusual for graves in this region and time period to contain more than one body [48,50,103]. The fact that at least ten people were identified among the retrieved assemblage, some with fatal head injuries, raises questions around the circumstances that led to their deaths and apparent burial in a communal grave. The skeletonised remains of these individuals are important because they record violent interaction between people who lived at a time when significant social and political imbalances existed between herders and hunter-gatherers.

Records dating back to the 1946 CE donation to UCT reported that the remains came from an antbear (*O*. *afer*) hole (S1 Fig). They noted that it seemed unlikely that all the individuals were buried at once in “the small hole", and that they might have been buried “at successive times in a family vault”. The records also speculate that they might have been victims of a smallpox epidemic. Smallpox in this region post-dates European contact [104], and in 1946 CE there was no way of knowing how long ago the Ladismith individuals lived. The idea of burial in a family vault was equally speculative. In 1946 CE, little was known of precolonial burial styles but today, based on recovery of several hundred individuals, we know that vaults (repeated interments in the same grave) did not occur in the precolonial Cape.

The burial was disturbed resulting in commingling and incomplete recovery of skeletal material; therefore, the true nature of burial remains speculative. However, the most parsimonious scenario is that the ten Ladismith individuals were buried in a communal grave, as observed elsewhere in this region [48,103,105]. Another possibility is a group of graves clustered very close together. In this region, groups of graves have occasionally been documented [52] or inferred [21], but where the positions of the graves are known, they are always separated by several metres–a pattern that would require extensive landscape disturbance to expose ten individuals. This was not the case, given that the 1946 CE records describe the hole as 1.4 m deep, 1 m long and 0.6 m wide (S1 Fig). The most likely interpretation of the Ladismith assemblage is that they derive from a single burial event in a communal grave that was subsequently disturbed by an antbear (or similar animal). Since all the individuals recovered are incomplete, it seems likely that the hole exposed only a portion of the burial. This disturbance would have scattered the skeletal elements, resulting in some being brought to the surface where they were noticed and collected. Some skeletal elements showed partial bleaching, indicating they were exposed on the surface for a period [106]. Exposed elements would have been easily scattered and destroyed by multiple taphonomic processes. This may explain why most individuals were represented only by larger, more robust skeletal elements. Ribs, vertebrae and smaller skeletal elements, including those of the hands and feet, were missing. The lack of crania is surprising, as human crania are readily recognised and if present, would likely have been recovered. However, if exposed on the surface they may have been collected at an earlier point in time, we cannot know. The uncertainties associated with radiocarbon measurements mean that radiocarbon cannot prove contemporaneity at the scale of the human lived experience. However, the three radiocarbon dates obtained for UCT 149, 151a and 157 are consistent with the hypothesis that these individuals perished in a single event.

The similarity of injuries observed in the crania of UCT 149, 150 and 151a suggests that they succumbed to similar violent fates. The blows to all three evidence purposeful violence, directed laterally in UCT 149, anteriorly to the base of the neck in UCT 150 and inferiorly through the entire cranial vault in UCT 151a. Similar cranial base injuries were observed in execution-type killings in the Khmer Rouge era of Cambodia [91,92]. The fracture on the left ulna of UCT 151a was likely a defensive injury sustained shortly before death. The injuries in UCT 149 indicate crushing against a surface, as would be the case if the child had been lying on their side when killed. There are no visible perimortem injuries on the adult (UCT 157) or adolescent (UCT 148) cranium, nor on any of the other post-crania.

Penetrating injuries previously described in Holocene hunter or herder crania mostly consist of circular, depressed lesions likely to have been inflicted by digging sticks or similar items [19; 48, S1 Fig; 49; 107]. At Melkbosstrand, just north of Cape Town, two individuals dating to *c*. 2500 BP show large gashes across their crania apparently made by an implement with uneven edges, resulting in ellipsoid or irregular lesions with internal bevelling. The authors suggest that the weapon was a flaked stone artefact [47]. At Lagoon Beach, also just north of Cape Town, an adult female dating to slightly less than 2000 years ago presented with extensive peri-mortem fracturing of the of the cranial vault and base as well as the front of the mandible and atlas vertebra [48]. The weapon used to inflict this blunt force trauma could not be identified. The morphologies of injuries in the Ladismith children are different from any previously described in the region. They suggest a slow loading impact by an object with straight and/or sharp edges. The dimensions of the injuries indicate a blade 20 mm to 50 mm wide and approximately 2 mm thick. The trauma on UCT 151a transects the entire cranial vault along the axial plane with no evidence of a hilt or handle penetrating the superior portion of the cranium. Thus, the blade exceeded 110 mm in length. These dimensions are inconsistent with a knife or arrow point, but consistent with a large, thin-bladed spear [108,109]. Similarities in the morphology and dimensions of cranial injuries (2 mm wide lesion on the parietal bone of UCT 149 and the uniform 2 mm notch defect observed in UCT 150) suggest that the same (or a very similar) weapon was used on at least these two individuals. In UCT 151a, there is extensive damage to the cranial vault and basicranium resulting from a forceful impact. Alignment of the superior and inferior injuries indicate that the weapon was long enough to penetrate both in a single blow.

At the time of these deaths, a variety of materials were available for manufacturing blades: bone, stone and metal. A bone implement was associated with the Ladismith burials. A drawing of this was included in the notes, describing it as a “Tsamma knife” (S1 Fig), an implement used to cut open the wild melons (*Citrullus lanatus*) that grow in dry parts of southern Africa. Unfortunately, the implement was unavailable for physical inspection at the time of writing. The smoothed edges illustrated in the drawing may be consistent with use as a cutting tool, but this does not account for the two holes near one end. A careful study of similar items from archaeological contexts suggests they are likely to be aerophone musical instruments, such as bullroarers [110,111]. Published descriptions of these instruments report dimensions ranging from 58–88.2 mm in length, 13–26.9 mm in width and 2–5 mm in thickness, with longer instruments having greater thickness [110]. A thin-bladed bone weapon estimated to be more than 100mm in length with a thickness of 2mm would have been too brittle to produce the injuries observed, especially in UCT 151a.

Bone and stone artefacts made by hunter/herder societies in this region and period are well known from excavated archaeological assemblages [112–114]. Hunters relied mainly on small bows and poisoned arrows. The arrows had bone or stone tips, intended primarily to pierce the skin and introduce the poison (typically placed just below the arrow tip) into the prey’s bloodstream. Death followed, sometimes several days later [42,115]. Spears were also used. These were probably made from wood, since at this time, people were not making finely-fashioned stone spear points, as found in much earlier times [116]. Lithic artefacts from nearby sites dating to the last 2000 years are extensively documented and described in several publications [112,117,118]. There are no thin-bladed (with edges 2 mm thick) stone or bone spear points from any of the archaeological assemblages in the region capable of inflicting the types of injuries observed on the Ladismith skeletons.

A flaked stone tool was suggested as a possible implement in the violent death of an adult female and adolescent from Melkbosstrand [47], mentioned above, resulting in large penetrating lesions with at least one irregular margin and internal bevelling. The straight edges of the lesions observed in the Ladismith children, characterised by minimal internal bevelling, particularly the narrow 2 mm-wide lesion on the parietal bone of UCT 149 and the uniform 2 mm notch defect in UCT 150, are inconsistent with injuries caused by stone implements. While the edge of a stone or bone implement may create a cut that is 2 mm thick, when penetrated to 110 mm as observed we would expect a more ellipsoid shaped injury with internal bevelling [119,120]. The observed injury margins are smooth and straight, consistent with sharp force injuries today made with metal composites, where the thin blades retain strength for deep penetration injuries. The presence of adhering bone scales in UCT 149 and 151a, and the uniform colour of the fracture margins and surrounded undamaged bone indicate that these lesions occurred perimortem [76], and therefore, exclude the possibility that they were created post-depositionally or by rough methods used during recovery (e.g. a spade).

In the absence of a weapon or implement present responsible for these injuries, interpretation is reliant on knowledge of the archaeological assemblages in the region, lesion morphology and forensic trauma analysis. Based on the evidence presented here we hypothesise that these broad, thin perforations were most likely inflicted with a metal implement(s), probably forged from iron. Additional analysis using scanning electron microscopy in the future may further elucidate the type and material of implement used [121–123].

**Fig 7:**
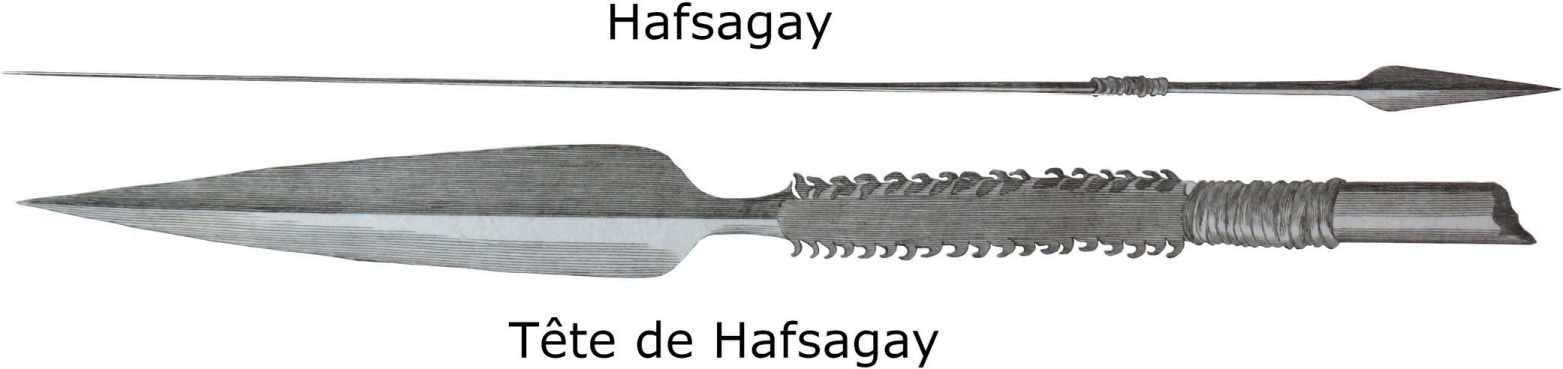
Drawings of an Assegaai (spear) observed by Sparrman in 1772 in the southern Cape [125].

Historical documents report the use of metal spears (assegaais) in the Cape region in the 18^th^ century CE. Although from a more recent period than the time of the Ladismith individuals, these reports may suggest the type of spears that would have been present. Thunberg in his travels of 1772 [124, p.85] described the presence of spears in the region of the present-day town of Wilderness, stating: “*The Hottentots* [herders] *always carry a javelin or two (assagays) with them on their journeys*. *These assagays consist of an iron point or head grooved on both sides along its whole length of six inches*. *It is sometimes round and smooth and sometimes barbed*. *This spear head is fastened with thongs of leather to a slender*, *round stick*, *six feet long*, *made of the assagay wood (*Curtisia faginea*)*, *and tapering towards the end*. *With these lances*, *which they throw with great dexterity to the distance even of 100 paces*, *they defend themselves against their enemies and wild beasts*, *and are able to kill with them*, *buffaloes*, *and other animals*.” *Curtisia faginea* is now called *Curtisia dentata*, and commonly referred to as the “assegai tree”. Similarly, Sparrman [125, p. 194] described spears observed on his travels in 1775, writing: “*The Hottentots who live in these parts*, *or within the boundaries of the Dutch colonies*, *seldom make use of any weapons*. *Here and there*, *indeed*, *a man will furnish himself with a javelin*, *by way of defense against the wolves*: *this is called a hassagai”* Sparrman’s illustration, reproduced in Fig 7, shows a weapon hypothetically capable of producing the injuries described above.

Iron-working was not practised in the Ladismith region in the precolonial period. As described above, local communities used implements and weapons made from stone, wood and bone. The nearest location where metal spears were produced was amongst iron-working farming communities at least 500 km away [126]. Permanent farming settlements were restricted to the summer rainfall regions of South Africa (see Fig 1), because their staple grain crops were sorghum, millet and later maize, all summer-rainfall dependent. The influence of these communities was, however, more extensive, especially through widespread trading networks. It is worth noting that the most south-eastern farming communities (those closest to Ladismith) were not rich in iron; even in later times, when iron was much more readily available, it was still sufficiently scarce that early 20^th^ century Xhosa, Thembu and Mpondo communities frequently made agricultural implements out of hard wood, rather than iron [127, pp.162-163; 128, pp.140-145; 129, pp.235,237]. In the Ladismith region in the early 15^th^ century CE, a metal spear would have been a valuable item.

**Fig 8:**
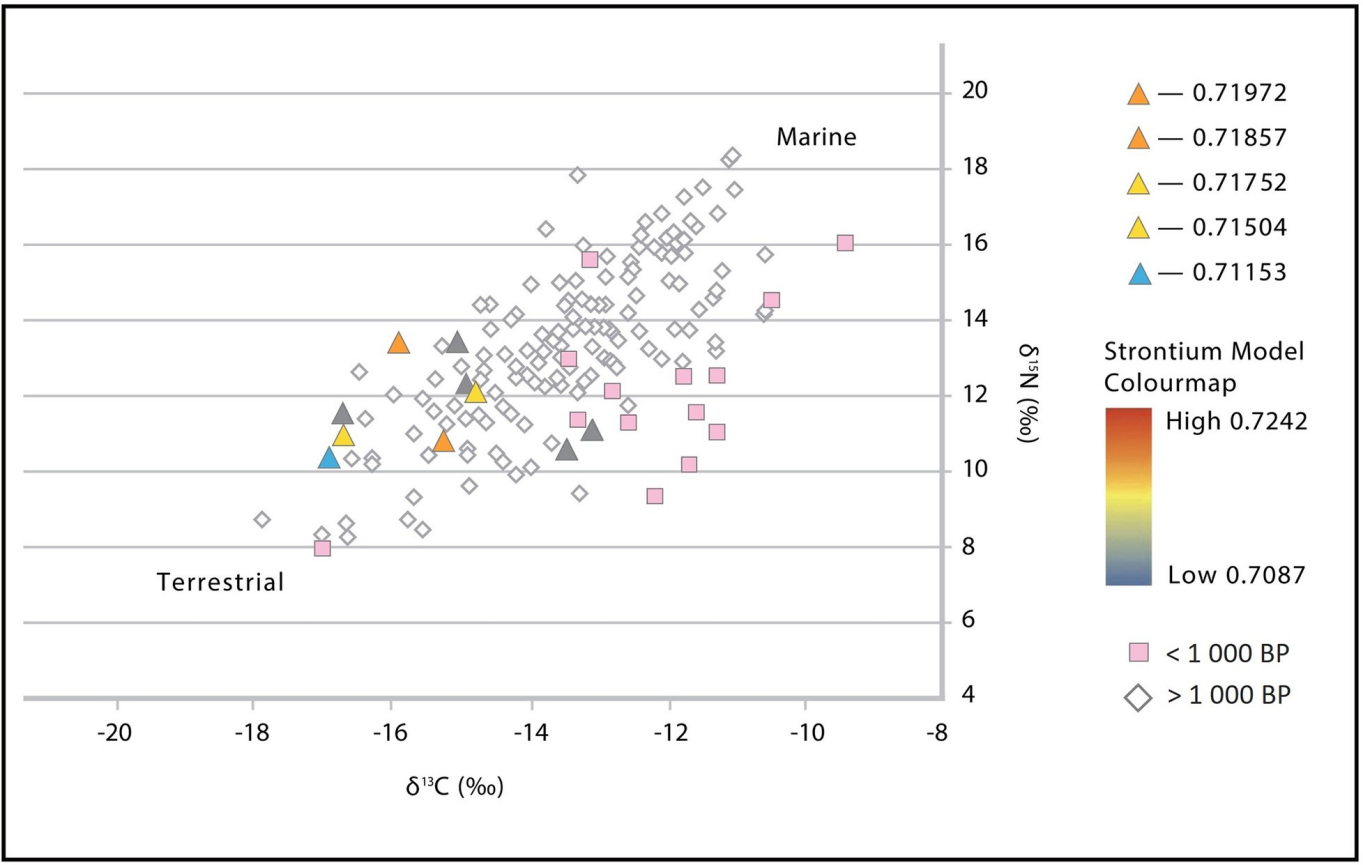
δ^15^N plotted against δ^13^C values for bone collagen of LSA skeletons recovered from the south coast and adjacent coastal plain of the Western Cape Province. Each symbol shows a different individual. Diamonds: >1 000 BP, Pink Squares: <1 000 BP, Triangles: Ladismith skeletons with ^87^Sr/^86^Sr heatmap. Grey triangles show individuals for whom we do not have ^87^Sr/^86^Sr values since teeth are not preserved. δ^13^C and δ^15^N values plotted for UCT 148 (lower orange triangle) are for bone.

Fig 8 shows the δ^13^C and δ^15^N values of the Ladismith individuals compared with other archaeological skeletons from the region (mostly from coastal localities). Individuals who plot towards the top right-hand side of the graph (high δ^13^C and δ^15^N) are coastal individuals who consumed a greater proportion of marine foods, whereas those who plot towards the bottom left (low δ^13^C and δ^15^N) consumed mainly terrestrial foods [99]. Individuals plotted as pink squares date to the second millennium CE (but pre-date European settlement in the region). Squares lying towards the right-hand side of the graph (low δ^15^N in combination with relatively positive δ^13^C values) show individuals who relied more on terrestrial C_4_-based foods and were probably cattle pastoralists consuming dairy products and beef [99].

The Ladismith individuals tend to plot in the lower halves of the ranges of both δ^13^C and δ^15^N, indicating that they consumed largely terrestrial diets. This is expected, given that Ladismith is approximately 100 km from the ocean. Since the bone and dentine samples had to be taken from different skeletal elements in different individuals, isotope measurements reflect diets at different times of life. Bone remodels throughout life [130,131], so its δ^13^C and δ^15^N values provide long-term averages of diet. Major load-bearing elements such as the femoral shaft remodel slowly, so that a proportion of tissue formed in adolescence is still present in later adulthood [132]. δ^13^C and δ^15^N values for long bone shafts (humerus from UCT 149, distal tibia from UCT 150, femur from UCT 151a and 152a, radius from UCT 152b and 152c), therefore, provide averages of diet consumed over a period of at least several years. When it was not possible to sample bone, or doing so would have involved an unacceptable degree of damage to an otherwise intact skeletal element (e.g. the mandible of UCT 152d), we measured δ^13^C and δ^15^N values in dentine. Values for dentine collagen are directly comparable to bone collagen in the sense that there are no metabolic offsets between the two [133,134], but dentine forms over a relatively short period of time and does not remodel (although there may be some subsequent addition of secondary and/or tertiary dentine). Its isotopic composition reflects diet at the time of dentine formation. Dentine samples were obtained from the tips of the tooth roots (see Methods) of the left mandibular M2 (for UCT 148 and 151b), the left mandibular M3 (for UCT 152d), and the left maxillary canine (for UCT 157). As discussed in the results, the values for UCT 148 reflect diet shortly before death. Those for UCT 151b reflect diet shortly before the time of death, since the root apex was incomplete. For UCT 152d, the root tip of the mandibular M3 probably reflects diet in this person’s early 20s [100], and for UCT 157, the root tip of the left maxillary canine reflects diet between 13.5–16.5 years of age [100]. UCT 152d is an older individual (Table 1), so dentine δ^13^C and δ^15^N values refer to a period perhaps two or more decades before death. UCT 157 was probably in his early 30s at the time of death (Table 1) so here too, dentine δ^13^C and δ^15^N reflect diet perhaps two decades before death.

Values for different individuals are widely scattered. UCT 150 and 152b lie towards the right-hand side of the distribution. They may have consumed cattle-derived foods, but since their values are somewhat marginal, we cannot be certain. The other Ladismith individuals have more negative δ^13^C values, indicating consumption of mainly C_3_-based foods, typical for hunter-gatherers in this region. The δ^13^C values of UCT 149, 152c and 152d, at -16.7 ‰, -16.7 ‰ and -16.9 ‰ respectively, are amongst the most negative in the entire dataset. UCT 157 is unusual in that the δ^15^N value (13.4 ‰) is high compared with δ^13^C (-15.9 ‰). This may reflect residence in a dry area, and/or greater consumption of animal-based foods. UCT 152c is an adult male, and UCT 149 an approximately 7-year-old juvenile. Given their very similar δ^13^C and δ^15^N values, and the degree of separation from other individuals (except UCT 152d), they consumed similar diets during their lifetime. Similarly, UCT 152a (adult male) and 151b (approx. 12-year-old juvenile) have identical (within analytical error) δ^13^C and δ^15^N (δ^13^C -14.8 ‰ and -14.9 ‰, δ^15^N 12.2 ‰ and 12.4 ‰) values. Overall, the δ^13^C and δ^15^N values observed in the ten individuals analysed extend across the entire range of variation observed in inland burials from the southern Cape. Even if we consider only values measured on bone collagen (not dentine), the pattern is one of substantial dietary diversity.

^87^Sr/^86^Sr values should be assessed against patterning across the landscape in this region, as reported by Copeland et al. [135]. These authors developed an “isoscape” (isotope map) of bioavailable ^87^Sr/^86^Sr for the Ladismith region, based on measurements of contemporary plants collected across the various geological substrates in the area. Values in the immediate vicinity of Ladismith are expected to be close to 0.719, and those near the coast to be lower; seawater [also shellfish, fish, marine mammals and other ocean-dwelling creatures] measures 0.709. Values of 0.71839 to 0.71972, as observed in UCT 148 and 157, match those of the Table Mountain Group substrates in the immediate vicinity of Ladismith, and to the north. For UCT 157, the range of values across the two teeth analysed extends from 0.71761 to 0.71919, a difference of 0.00158. This individual moved around during the period of tooth formation, but within the area underlain by the geologically ancient rocks of the Cape Fold Belt [135]. The value of 0.71504 observed in UCT 149 is not local, and probably reflects residence in the region to the south. Most striking is the value of 0.71153 for UCT 152d, indicating that during the formation of the 3rd molar crown (*c*. 8–14 years), this individual lived on the coastal plain, a minimum of 80 km south of Ladismith. Their negative δ^13^C (-16.9 ‰) and low δ^15^N (10.3 ‰) values indicate a mainly terrestrial diet. We may infer that this person consumed mainly terrestrial plant and animal foods obtained on the coastal plain. Overall, the degree of ^87^Sr/^86^Sr variation between the individuals in this study shows that they spent their early lives in regions with different geological substrates.

## Conclusions

We infer that the Ladismith group consisted of a diverse band of at least five adults, an adolescent and four children. At this stage, we do not know what familial relationships, if any there may have been, but since three of the children were aged 5–7 years, they were likely accompanied by caregivers. Future research (e.g. aDNA) may elucidate this. Members of the group had lived in different regions during their early lives (recorded in enamel ^87^Sr/^86^Sr) and consumed very different diets but had banded together and were living in or travelling through the Ladismith area at the time of their deaths. Their δ^13^C and δ^15^N values, especially those that plot towards the lower left-hand side of Fig 8 are typical of hunter-gatherer diets in this region. The most parsimonious explanation for multiple children exhibiting clear evidence of similar fatal injuries, followed by burial in a communal grave, is that they perished in a single violent confrontation with stronger adversaries. Based on the dimensions of the cranial lesions, we infer that those adversaries were armed with one or more iron spears, weapons probably obtained through long-distance trade. It is unclear if the other individuals succumbed to the same fate. Given the local context, it is plausible that this event may have arisen from a conflict between hunters and herders, although definitive confirmation remains elusive. If iron weapons were indeed used, the implication is that Bantu-speaking farming communities in the east and herding/hunting societies in the Western Cape were more closely interconnected than previously realised.

## Supporting information

S1 Fig Entry in the accessions register of the Human Skeletal Repository regarding the Ladismith skeletons and drawing of the bone implement associated with them. This was not available for visual inspection at the time of this study. Rather than a knife, it may be an aerophone (a musical instrument that produces audible vibrations by displacing air) [110,111]. The initials M.R.D. stand for Matthew Robertson Drennan, Professor of Anatomy at the University of Cape Town in the 1940s.
(TIF)

S2 Fig Inventory diagrams of all Ladismith individuals. Elements in grey are present.
(TIF)

S3 Fig Ladismith crania. (TIF)

S1 File Dental inventory. (DOCX)
