# Supplementary figures and images for: Bioarchaeology and evidence of violence from a precolonial later stone age communal burial in South Africa
Source: PLoS One. 2024 Sep 17;19(9):e0310421. doi: 10.1371/journal.pone.0310421 (PMC11407628; doi:10.1371/journal.pone.0310421)

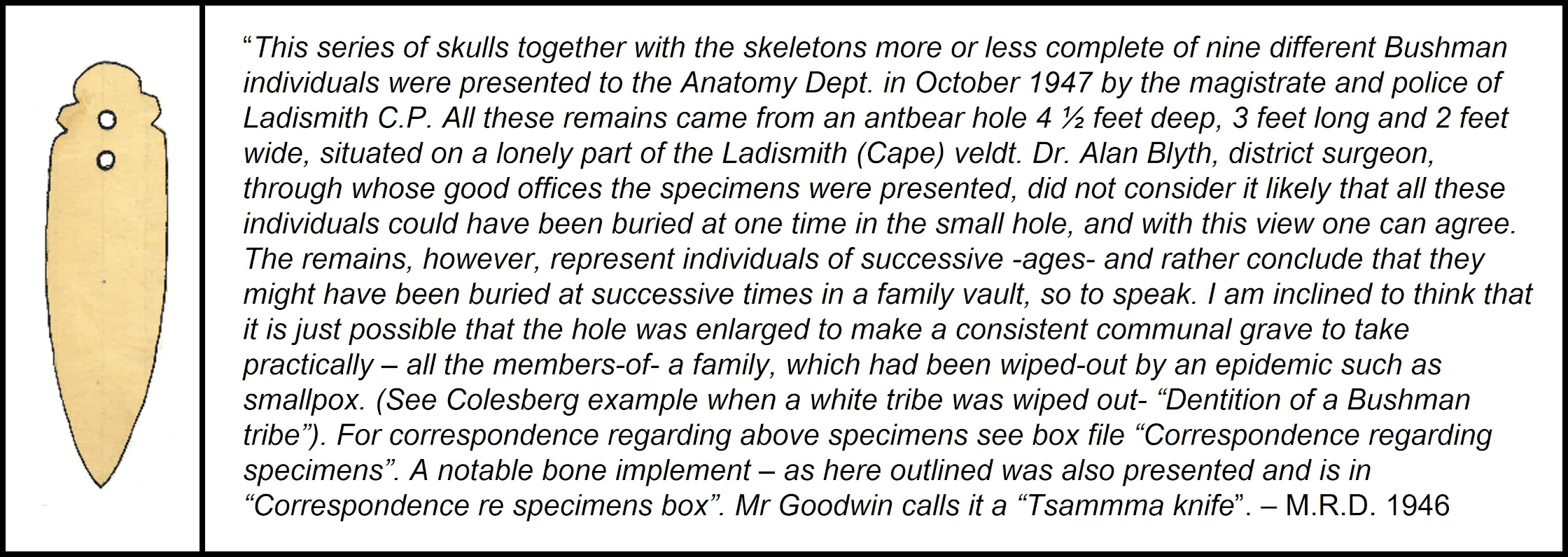

Supplement: S1 Fig — This was not available for visual inspection at the time of this study. Rather than a knife, it may be an aerophone (a musical instrument that produces audible vibrations by displacing air) [110,111]. The initials M.R.D. stand for Matthew Robertson Drennan, Professor of Anatomy at the University of Cape Town in the 1940s. (TIF) [file pone.0310421.s001.tif]

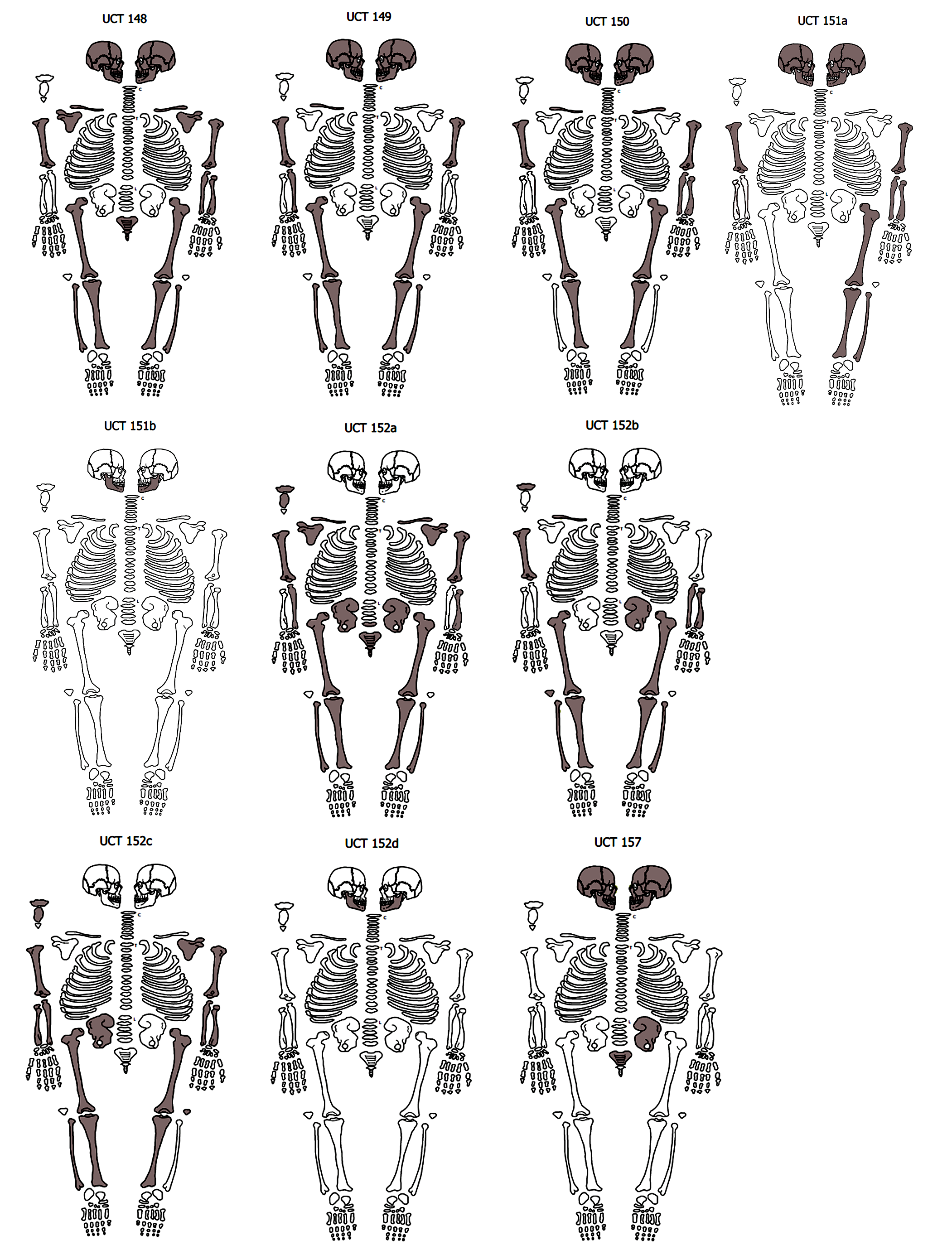

Supplement: S2 Fig — Elements in grey are present. (TIF) [file pone.0310421.s002.tif]

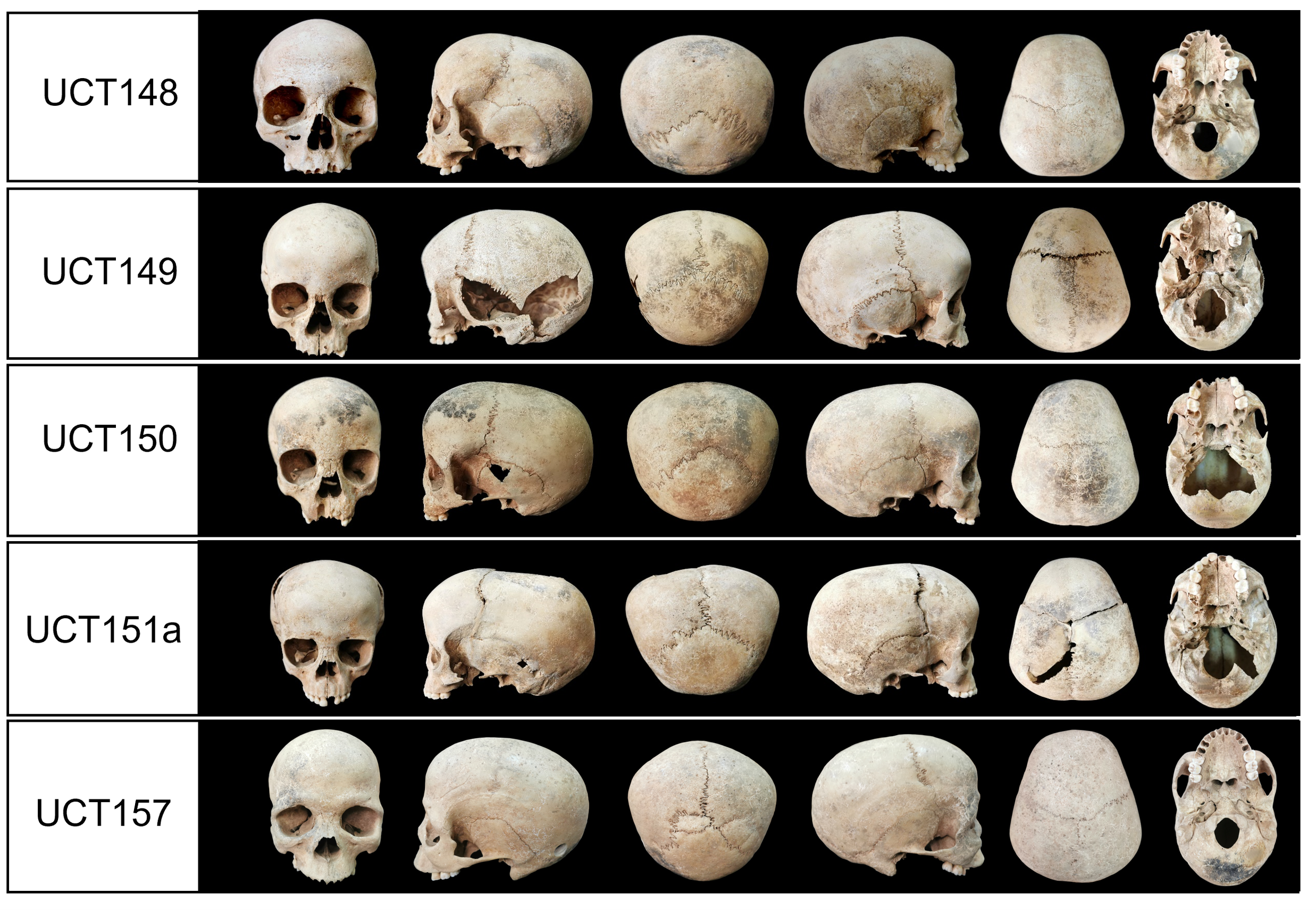

Supplement: S3 Fig — (TIF) [file pone.0310421.s003.tif]
