# Supplementary material for: Bioarchaeology and evidence of violence from a precolonial later stone age communal burial in South Africa
Source: PLoS One. 2024 Sep 17;19(9):e0310421. doi: 10.1371/journal.pone.0310421 (PMC11407628; doi:10.1371/journal.pone.0310421)
Supplement: S1 File — (DOCX) [file pone.0310421.s004.docx]

**S4 File: Dental inventory**

For dental inventory the FDI World Dental Federation notation (also known as “FDI notation” or “ISO 3950” notation) was used. Developed by the World Dental Federation, this notation system is commonly used worldwide and has been designated by the International Organization for Standardization as standard ISO 3950 "Dentistry — Designation system for teeth and areas of the oral cavity" (ISO, 2016). The system uses a two number system for the location and naming of each tooth. Each tooth is represented by a two-digit number, in which the first digit indicates the tooth quadrant and if it is permanent or deciduous, and the second digit indicates the tooth type.

According to this system, the jaw is divided into four quadrants between the central incisors and the upper and lower dental arches. The first number refers to the quadrant of a tooth: 1 = right upper quadrant; 2 = left upper quadrant; 3 = left lower quadrant; 4 = right lower quadrant. The second number refers to the individual tooth within a specific quadrant: 1 = central incisor; 2 = lateral incisor; 3 = canine; 4 = 1st premolar; 5 = 2nd premolar; 6 = 1st molar; 7 = 2nd molar; 8 = 3rd molar.

In deciduous teeth the system is similar however in describing the quadrants the numbers 5 to 8 are used in the place of 1 to 4 to designate quadrants. Similarly, due to the absence of the premolars in deciduous dentition the numbers 1 to 5 are used only, to indicate the central incisor, lateral incisor, canine, 1st molar and 2nd molar respectively.

**Inventory list** (list of teeth that are present)**:**

**UCT 148:** 16, 17, 18 (unerupted crown), 26, 27, 37, 38, 46, 47, 48.

**UCT 149:** 11, 12, 13, 14, 17, 21, 22, 23, 24, 25, 26, 27, 31, 32, 33, 35, 37, 41, 42, 43, 45, 46, 47, 64, 65, 74, 84. [Adult teeth were unerupted except 26, 46].

**UCT 150:** 12, 17, 21, 22, 27, 31, 32, 37, 41, 42, 47, 54, 55, 63, 64, 65, 75, 84, 85. [Visible adult teeth were unerupted].

**UCT 151a:** 16, 26, 36, 37, 46, 47, 51, 52, 53, 54, 55, 62, 63, 64, 65, 72, 74, 75, 81, 82, 84, 85. [Visible adult teeth were unerupted].

**UCT 151b:** 35, 36, 37, 42, 43, 44, 46, 47 [Mandible only].

**UCT 152a:** No dentition

**UCT 152b:** No dentition

**UCT 152c:** No dentition

**UCT 152d:** 31,34, 36, 38, 41 **[**Mandible only**]**

**UCT 157:** 16, 17, 18, 23, 26, 27, 28, 34, 36, 37, 38, 44, 46, 47, 48.

**References:**

ISO (International Organization for Standardization). *ISO 3950:2016 Dentistry — Designation system for teeth and areas of the oral cavity*, Edition 4. 2016. Available from: <https://www.iso.org/standard/68292.html>
